# The Redox Proteome of Thiol Proteins in the Rice Blast Fungus *Magnaporthe oryzae*

**DOI:** 10.3389/fmicb.2021.648894

**Published:** 2021-03-10

**Authors:** Xinrong Zhang, Zhenhua Zhang, Xiao-Lin Chen

**Affiliations:** ^1^State Key Laboratory of Agricultural Microbiology, Provincial Key Laboratory of Plant Pathology of Hubei Province, College of Plant Science and Technology, Huazhong Agricultural University, Wuhan, China; ^2^State Key Laboratory of Agrobiotechnology, Ministry of Agriculture Key Laboratory for Plant Pathology, China Agricultural University, Beijing, China; ^3^Department of Genetics, University Medical Center Groningen, Groningen, Netherlands

**Keywords:** post-translational modification, *Magnaporthe oryzae*, redox proteome, thiol proteins, fungal infection, oxidative stress

## Abstract

Redox modification, a post-translational modification, has been demonstrated to be significant for many physiological pathways and biological processes in both eukaryotes and prokaryotes. However, little is known about the global profile of protein redox modification in fungi. To explore the roles of redox modification in the plant pathogenic fungi, a global thiol proteome survey was performed in the model fungal pathogen *Magnaporthe oryzae*. A total of 3713 redox modification sites from 1899 proteins were identified through a mix sample containing mycelia with or without oxidative stress, conidia, appressoria, and invasive hyphae of *M. oryzae*. The identified thiol-modified proteins were performed with protein domain, subcellular localization, functional classification, metabolic pathways, and protein–protein interaction network analyses, indicating that redox modification is associated with a wide range of biological and cellular functions. These results suggested that redox modification plays important roles in fungal growth, conidium formation, appressorium formation, as well as invasive growth. Interestingly, a large number of pathogenesis-related proteins were redox modification targets, suggesting the significant roles of redox modification in pathogenicity of *M. oryzae*. This work provides a global insight into the redox proteome of the pathogenic fungi, which built a groundwork and valuable resource for future studies of redox modification in fungi.

## Introduction

Protein features and functions are modulated not only by sequence of the amino acids but also by post-translational modifications (PTMs), such as acetylation, lipidation, methylation, and oxidation–reduction modification (as known as redox modification). PTMs are ideal approach to maintain the cell homeostasis and response to external or internal stimuli by recasting the function, stability, or activity of translated proteins, which consequently impacts the organism at different levels such as genome and epigenome (Go and Jones, 2013). In PTMs, the redox modification on cysteine residues is an important source of regulations on protein functions (Go and Jones, 2013; Sievers et al., 2018), which impacts many physiological pathways and biological processes (Zhao and Jensen, 2009; Baraibar and Friguet, 2013; Olsen and Mann, 2013).

The reactive thiol groups attached to cysteine residues are responsible to the redox modifications by shifting from reduction to oxidation or conversely (Winterbourn and Hampton, 2008; Hancock, 2009; Paulsen and Carroll, 2010), where the shifting is either irreversible (e.g., sulfonic acid) or reversible (e.g., disulfide bonds) (Murray and Van Eyk, 2012). In previous studies, it has been revealed that protein structures and functions can be transformed by reversible redox modifications (D’Autreaux and Toledano, 2007; Heppner et al., 2018; Su et al., 2019). However, the consequence of redox modification on proteins varies depending on both protein itself (e.g., biochemical properties and three-dimensional arrangement) and reactive oxygen species (ROS) (e.g., abundance and species) (Corcoran and Cotter, 2013), which introduces complexes and uncertainties to redox proteome.

Reactive oxygen species are well known as by-products of metabolism and internal source of oxidants in cells (Corcoran and Cotter, 2013) and are harmful to biomolecules if they are overaccumulated, which can eventually be toxic to cells, tissues, and organisms. As another aspect of the two-side sword, ROSs, such as hydrogen peroxide, have been demonstrated as mediators in redox signaling pathways by activating redox modifications to particular target molecules (Bae et al., 2011). Of all the underlying mechanisms that ROS mediates redox signaling, reversible oxidation of proteins with cysteine residues, known as thiol proteins, is well established to trigger and transmit redox signals, which are related to many critical biological activities such as proliferation (Darnell et al., 1994), stress response (Sies, 2014, 2017), and apoptosis (Tobiume et al., 2001; Giorgio et al., 2005; Thangima Zannat et al., 2011).

Redox modifications are widely present in eukaryotes and prokaryotes and involve wildly in multiple biological process and signal pathways. Quantified investigations on the thiol redox proteome have been studied on bacteria (*Escherichia coli*) (Xie et al., 2019), algae (*Chlamydomonas reinhardtii*, *Phaeodactylum tricornutum*, cyanobacterium *Synechocystis*, cyanobacterium *Prochlorococcus*) (McDonagh et al., 2012; Guo et al., 2014; Rosenwasser et al., 2014; McConnell et al., 2018), nematodes (*Caenorhabditis elegans*) (Kumsta et al., 2011), and plants (*Arabidopsis thaliana, Triticum aestivum*) (Bykova et al., 2011; Liu et al., 2014). However, the redox modification of proteome in fungi has not yet been identified so far, so that it is interesting to know about the extent and function of redox in fungi.

*Magnaporthe oryzae* is a filamentous fungus that causes rice blast, one of the most destructive diseases of cultivated rice and threatens worldwide food production (Wilson and Talbot, 2009; Dean et al., 2012). *M. oryzae* is a model fungus that is used to study the physiological and pathogenic molecular mechanism of the plant pathogenic fungi (Wilson and Talbot, 2009). It has been reported that intricate physiological redox balance is essential for the pathogenicity of *M. oryzae*, such as functional appressorium formation and penetration (Kou et al., 2019). In order to determine roles of the redox modification in fungi, in this study, the thiol-oxidized proteome analysis was performed in *M. oryzae*, through a mass spectrometry (MS)-based strategy. A global landscape of the redox modification sites on protein was shown, providing insights into functions of redox modification in the pathogenic fungi, which also built a groundwork for redox modification studies in other pathogenic or non-pathogenic fungi.

## Materials and Methods

### Strains and Cultural Conditions

The wild-type strain of *M. oryzae* P131 used in this study was maintained on Oatmeal Tomato Agar (OTA) plates at 28°C. Mycelia were incubated in liquid CM cultured on a rotary shaker (180 rpm) for 36 h at 28°C. Conidiation, appressorium formation, and inoculation on barley were performed as described previously (Chen et al., 2014, 2020).

### Preparation of Protein Samples

A mixture of mycelia, conidia, appressorium, infection hyphae, and mycelia treated with H_2_O_2_ was used for protein extraction and liquid chromatography–tandem mass spectrometry (LC-MS/MS) Analysis. Mycelia samples were harvested from liquid CM after shaking culture for 36 h. For H_2_O_2_-shocked mycelia samples collection, 10 μM H_2_O_2_ was added to the CM medium after the mycelia was shaking cultured in liquid CM for 36 h, and then, the mycelia were cultured for another 12 h for harvest. Conidia samples were collected after conidiation as described above. Appressorium sample collection was performed as described previously (Chen et al., 2020). For infection hyphae collection, conidia suspension (1 × 10^5^ conidia/mL) was sprayed on the lower leaves of 1-week-old barley and then incubated in a dark, moist chamber at 28°C. The lower barley epidermis together with the infection hyphae on the epidermis were torn down after 48 hpi. All the samples described above were stored at –80°C before processing. Lysis buffer (8 M urea, 1% Triton-100, 10 mM dithiothreitol, and 1% protease inhibitor cocktail) was added to the grinded samples, followed by sonication, refrigerated centrifugation, precipitation, and washing. The protein was redissolved in 8 M urea and quantified with bicinchoninic acid (BCA) method.

### High-Performance Liquid Chromatography Tandem Mass Spectrometry Analysis

#### IodoTMT (Thermo Fisher Scientific, United States) Labeling

Six volumes of cold acetone per 100 μg protein was added to the protein samples followed by precipitation at –20°C for 4 h. Protein was redissolved with 100 μl HES buffer [50 mM 4-(2-hydroxyethyl)-1-piperazineethanesulfonic acid (HEPES), pH 8.0, 1 mM ethylenediaminetetraacetic acid (EDTA), 0.2% sodium dodecyl sulfate (SDS)] and processed according to the manufacturer’s protocol for iodoTMT kit. Briefly, tris(2-carboxyethyl)phosphine (TCEP) was added to the sample to 10 mM and incubated at 37°C for 1 h and then mixed with the labeling reagent, which dissolved in methyl alcohol. After incubated in darkness for 1 h at 37°C, six volumes of cold acetone was added to the mixture followed by precipitation at –20°C for 4 h.

#### Protein Trypsin Digestion

Trypsin was added at 1:50 trypsin-to-protein mass ratio for the first digestion overnight and at 1:100 trypsin-to-protein mass ratio for a second 4 h digestion at 37°C. The peptides were then desalted and dried by vacuum freeze drying.

#### HPLC Fractionation

The tryptic peptides were fractionated into fractions by high pH reverse-phase high-performance liquid chromatography (HPLC) using Thermo Betasil C18 column (5 μm particles, 10 mm ID, 250 mm length) (Thermo Fisher Scientific, United States). Briefly, peptides were first separated with a gradient of 8–32% acetonitrile (pH 9.0) over 60 min into 60 fractions. Then, the peptides were combined into four fractions and dried by vacuum freeze drying.

#### Enrichment

Peptides dissolved in IP buffer (100 mM NaCl, 1 mM EDTA, 50 mM Tris–HCl, 0.5% NP-40, pH 8.0) were incubated with prewashed Anti-TMT antibody beads (Lot number Prod#90076, Thermo Fisher Scientific, United States) at 4°C overnight with gentle shaking. Then, the beads were washed four times with IP buffer and twice with deionized water. The bound peptides were eluted from the beads with 0.1% trifluoroacetic acid, and the eluted fractions were combined and vacuum freeze dried. The resulting peptides were desalted with C18 ZipTips (Millipore, United States).

#### LC-MS/MS Analysis

The peptides were dissolved in solvent A (0.1% formic acid and 2% acetonitrile) and loaded onto a reversed-phase analytical column. The gradient was comprised of an increase from 8 to 25% solvent B (0.1% formic acid and 90% acetonitrile) over 24 min, 25 to 35% in 8 min and climbing to 80% in 4 min, then holding at 80% for the last 4 min, all at a constant flowrate of 500 nl/min on an EASY-nLC 1000 UPLC system (Thermo Fisher Scientific, United States).

The peptides were subjected to NSI source followed by MS/MS in Q ExactiveTM Plus (Thermo Fisher Scientific, United States) coupled online to the ultraperformance liquid chromatography (UPLC). The electrospray voltage applied was 2.1 kV. The m/z scan range was 350–1800 for full scan, and intact peptides were detected in the Orbitrap at a resolution of 70,000. Peptides were then selected for MS/MS using NCE setting as 28, and the fragments were detected in the Orbitrap at a resolution of 17,500, a data-dependent procedure that alternated between one MS scan followed by 20 MS/MS scans with 15.0 s dynamic exclusion. Automatic gain control (AGC) was set at 5E4. Fixed first mass was set as 100 m/z.

### Bioinformatic Analysis

#### Search Protein Sequence Against Database

We processed all the raw MS data using MaxQuant software (version 1.5.2.8) (Tyanova et al., 2016). The parameters were set as the following. *M. oryzae* strain 70-15 including 12,991 sequences were use as the reference database including two decoy databases (an artifact database to calculate the false discovery rates caused by random matching and a contamination protein database to remove adulterate proteins). Corresponding to the enzyme digestion procedure, Trypsin/P was chosen as cleavage enzyme with up to four missing cleavages. In the two search scenarios, mass tolerance for precursor ions of First search and Main search were adapted to 20 and 5 ppm, respectively, while we set the mass tolerance for fragment ions as 0.02 Da. As to modification, carbamidomethyl on cysteine was considered as fixed modification, while alkylation on cysteine residues, oxidation on methionine residues, and n-terminal acetylation were accounted as variable modifications. We use FDR < 0.01 as adjusted significant threshold, and the minimum score for modification peptides was 40.

#### Sequence and Motif Analysis

The motif analysis was conducted by MoMo (version 5.0.2) (Cheng et al., 2019) application. A 21-AA window center to the modification sites was use to scan motifs. All the rest parameters were default except that the minimum occurrences was set to 20.

#### Protein Domain Analysis

Redox-modified proteins (RMPs) were annotated by InterProScan (Quevillon et al., 2005), an online tool based on sequence alignment method, against InterPro domain database.

#### Function Annotation and Enrichment Analysis

In GO analysis, the identified RMPs were mapped to UniProt-GOA (Huntley et al., 2015) database to get the corresponding UniProt IDs; then, the UniProt IDs were mapped to GO IDs. For RMPs without a UniProt annotation, we used InterProScan to assign GO terms to the proteins. After pooling both set of annotations, the proteins were classified by three GO categories: biological process, cellular component, and molecular function. In Kyoto Encyclopedia of Genes and Genomes (KEGG) pathway analysis, we used KASS (KEGG online service tools) to annotate RMPs, and the outcome annotations were mapped to KEGG pathway database by another online tool KEGG mapper. The subcellular localization of RMPs was conducted by the online tool wolfpsort (Horton et al., 2007). The enrichment of assigned functions was estimated using Fisher’s exact test with all proteins in *M. oryzae* as background, and the enriched cluster with p < 0.05 was considered as significant.

#### Protein–Protein Interaction Analysis

We selected 425 proteins with functional annotations (i.e., excluding uncharacterized or hypothetical proteins) and stringent quality control thresholds [Maxquant score > 80, –1.5 ≤ mass error (ppm) ≤ 1.5] to construct protein–protein interaction (PPI) network for proteins with redox modifications. The PPI network was obtained from STRING (version 11) (Szklarczyk et al., 2019) database with “confidence score” > 0.7 (high confidence) “active interaction sources” limited to Experiments, Database, Coexpression, and Neighborhood. The figure visualizing the network was constructed by Cytoscape (version 3.8.0) with plugin stringApp (version 1.5.1). To achieve a high confident PPI network, the final confidence score threshold of the PPI network was set to 0.9.

## Results

### Identification of Thiol Proteome in *M. oryzae*

**FIGURE 1:**
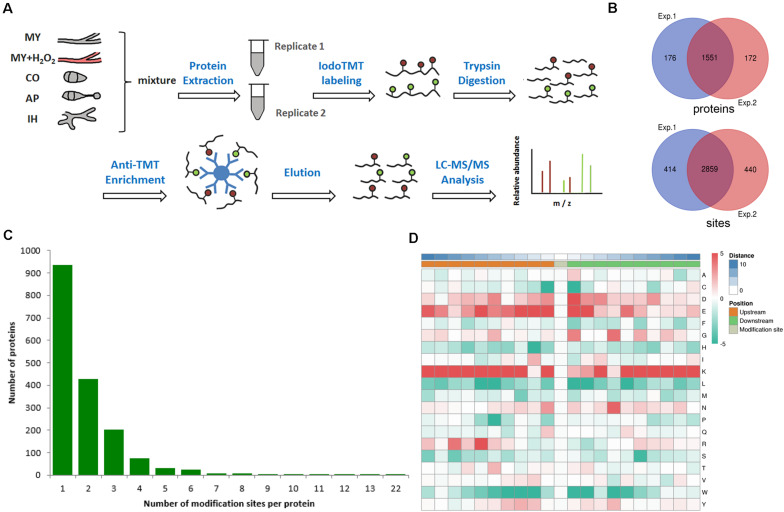
Redox proteomics identification of thiol-modified proteins in *M. oryzae*. **(A)** Flow chart of the integrated strategy for quantitative analysis of the redox proteome. **(B)** Venn diagram of modified proteins identified in replicates. **(C)** Venn diagram of modified sites identified in replicates. **(D)** The motif enrichment heatmap of upstream and downstream amino acids of all identified modification sites. Red and green indicate that this amino acid is more and less abundant close to the modification site.

To identify the redox sites in *M. oryzae*, we performed the proteomic survey of thiol using LC-MS/MS analysis in combination with immunoaffinity enrichment. In order to identify as many redox peptides as possible, we gathered samples from mycelia, conidia, appressorium, infection hyphae of *M. oryzae*, as well as mycelia shocked with reactive oxygen species. The experimental strategy is depicted in Figure 1A. In the first repeat, 3273 sites from 1727 proteins were identified while 3299 sites from 1723 proteins in the second repeat. In total, 3713 redox sites belonging to 1899 proteins have been identified, while 2859 redox sites from 1551 proteins were identified by both two repeats, as shown in Figure 1B and Supplementary Table 1. This result indicated that near 15% of the predicted *M. oryzae* proteins (around 13,000) can be modified by thiol redox, in at least one of the testing samples. The identified redox sites and the corresponding proteins were analyzed from different aspects to shed a light to the redox proteome in *M. oryzae.*

### Patterns of the Redox Modified Sites and Proteins

Among the identified thiol redox-modified proteins, around 50% had only one thiol-modified site, and 22, 10, or 3.2% had two, three, or four thiol modified sites, respectively. Other proteins had more than four thiol-modified sites, even up to 22 sites (Figure 1C). To discover the common features shared by proteins containing redox modification sites, amino acids motifs besides the modification sites were identified. The normal pattern of the motifs could indicate important function hub in respect to evolutionary conservation; therefore, we calculated the abundance of amino acids around the redox modification sites. The redox modification-specific motifs were discovered by MoMo Tools, which is a particular effort to characterize post-translational modifications in proteins. The detected motifs show the amino acids abundance around the redox modification sites (Figure 1D). By the abundance analysis, we found both positively and negatively charged amino acids (i.e., lysine and arginine; glutamic acid and aspartic acid) overrepresented in a 21-residues wide window centered to the redox modification site. However, when it comes to motifs, only positively charged residues are examined as principle conserved elements. In addition, the residues are mainly located in the upstream, which is at least three residues away from the modification sites. These findings suggest that redox reactive modification actively happens to lysine-rich segments and basic polar amino acids regions (Figure 1D). The corresponding three-dimensional protein structures and biochemical properties derived from the motifs could manipulate the sensitivity of thiols/disulfides in *M. oryzae*.

**FIGURE 2:**
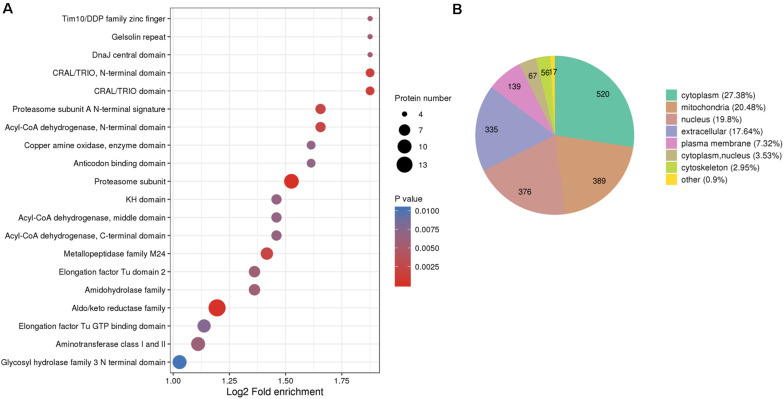
Protein domain enrichment and subcellular localization of the identified thiol proteins. **(A)** Protein domain enrichment bubble plot of proteins corresponding to modification sites. **(B)** Subcellular localization chart of proteins corresponding to modification sites.

Protein domains, by definition, are conservative amino acids sequences, the length of which vary between 25 and 500 amino acids, shared by various protein molecules repeatedly. The shared sequences are basic evolutionary components and usually function and structure similarly. To explore the evolutionary conservation of all identified RMPs, we estimated the enrichment of protein domains. The top 5 enriched domains are “Tim10/DDP family zinc finger” (Jarosch et al., 1996; Paschen et al., 2000), “Gelsolin repeat” (Silacci et al., 2004), “Dnaj central domain” (Qiu et al., 2006), “CRAL/TRIO, N-terminal domain” (Panagabko et al., 2003), and “CRAL/TRIO domain” (Panagabko et al., 2003; Figure 2A). In brief, the leading domains function as switcher that control mitochondrial protein import (Jarosch et al., 1996; Paschen et al., 2000), as organizer that control actin arrangement (Silacci et al., 2004), as chaperones that protect proteins (Qiu et al., 2006), and as modulators that mediate interaction between retinoids and visual cycle enzyme (Panagabko et al., 2003).

The translocation of a protein determines the exact locus in which the protein fulfills its mission consequently; therefore, localization of proteins at the subcellular level could benefit, for example, the identification of drug targets. However, the aberration of protein translocation endangers the homeostasis of organisms. Moreover, the controlled distribution of redox proteome is pivotal to intracellular physiological balance; for instance, redox processes are well regulated in mitochondria to protect cellular components (Rigoulet et al., 2011). Therefore, the generalized subcellular localization of RMPs contributes to our understanding on redox proteome in *M. oryzae*. To give an insight into the distribution of RMPs at the subcellular level, we predicted the subcellular localization of identified redox proteins and further classified them based on the assigned loci. In Figure 2B, the identified proteins distributed in different locations and the major four predictions are cytoplasm (27.38%), mitochondria (20.48%), nucleus (19.8%), and extracellular (17.64%), suggesting that the redox-modified proteins are widely distributed and play distinct roles in different subcellular regions of the cell.

### Thiol Redox Modification Is Involved in Many Cellular Processes and Metabolisms

**FIGURE 3:**
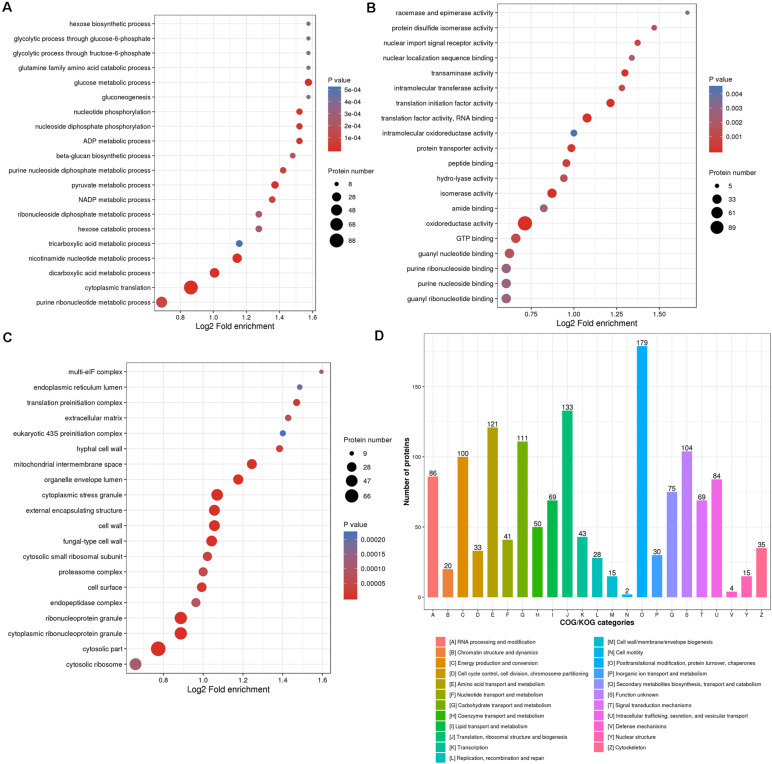
Enrichment categories of the identified thiol proteins. Gene Ontology (GO) classification enrichment bubble plots of the thiol proteins were shown based on **(A)** biological processes, **(B)** cellular component, and **(C)** molecular functions. **(D)** Clusters of orthologous groups functional classification chart of the thiol proteins.

For the goal to disclose how proteins undergoing redox reaction contribute to the various biological processes in *M. oryzae*, proteins identified with redox modification sites in this study were annotated by Gene Ontology (GO). Considering the informative difference across GO categories, level 2 GO annotations were chosen to represent features and characteristics of proteins that potentially perform important roles in different biological processes (BP), cellular components (CC), and molecular functions (MF) (Supplementary Table 2 and Figures 3A–C).

All of the identified RMPs were assigned level 2 GO terms using the KEGG mapper (Kanehisa and Sato, 2020), and an enrichment analysis was performed to exhibit the overrepresentation of terms (including but not limited to the level 2) related to redox proteome in *M. oryzae*. As shown in Figure 3A, regarding biological processes, RMPs are mostly enriched in nutrient utilization processes, including hexose biosynthetic process, glycolytic processes through glucose-6-phosphate and fructose-6-phosphate, glutamine family amino acid metabolic process, glucose metabolic process, hexose metabolic process, gluconeogenesis, as well as tricarboxylic acid metabolic process. This result suggested that redox modification plays key roles in maintaining cellular processes for fungal survival and development. Many other cellular processes such as nucleotide phosphorylation, ADP and nicotinamide adenine dinucleotide phosphate (NADP) metabolic processes, beta-glucan biosynthetic process, purine and pyruvate metabolic processes, cytoplasmic translation, etc. were also significantly enriched (Figure 3A), indicating widespread functions of redox modification in cellular processes.

The mostly enriched categories in molecular functions included racemase and epimerase activity, protein disulfide isomerase activity, intramolecular oxidoreductase activity, amide binding, oxidoreductase activity, and GTP binding activity, which were well consistent with the original function of redox modification (Figure 3B). Besides, the redox modification may also affect activities of nuclear import signal receptor, nuclear localization sequence binding, intracellular transferase, translational initiation factor, RNA binding, protein transporter, peptide binding, hydrolyase, isomerase, guanyl nucleotide and ribonucleotide binding, and purine nucleoside and ribonucleoside binding (Figure 3B), also suggesting a widespread role of the redox modification in regulating protein functions.

Categories belonging to cellular components were mostly enriched in multi-eIF complex, which reflects its role of oxidoreduction (Figure 3C). The translation-related complexes, such as endoplasmic reticulum lumen, translation preinitiation complex, eukaryotic 43S preinitiation complex, cytosolic small ribosomal subunit, and cytosolic ribosome were also significantly enriched, suggesting a regulatory role of redox modification in translation (Figure 3C). Other enriched cellular components included cell wall and extracellular component (extracellular matrix, hyphal cell wall, external encapsulating structure, cell surface, etc.), cytoplasmic stress granule, organelle envelope lumen, proteasome complex, endopeptidase complex, and ribonucleoprotein granule (Figure 3C).

In sum, GO annotations could demonstrate the common features across the whole redox proteome and shed a light on the underlying mechanisms of redox-related biological processes and molecular mechanisms in *M. oryzae*.

To understand the function of identified RMPs, we also grouped them into Clusters of Orthologous Groups of Proteins (COG) (Supplementary Table 2; Tatusov et al., 2000). The orthologous groups are known as conserved clusters sharing identical or analogous biological functions and biochemical properties. As shown in Figure 3D, the function clusters are mainly associated with the following: (i) (post-)translation and (post-)transcription including “post-translational modification, protein turnover, chaperones,” “translation, ribosomal structure and biogenesis,” “RNA processing and modification;” (ii) metabolism such as “amino acid transport and metabolism,” “carbohydrate transport and metabolism,” and “energy production and conversion.” These results were well consistent with GO annotation of biological processes, cellular components, and molecular functions.

### Metabolic Pathway Analysis of RMPs

**FIGURE 4:**
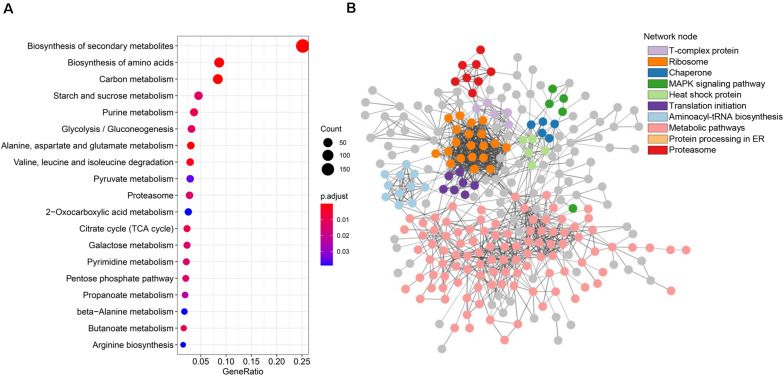
Metabolic pathway and protein–protein interaction analyses of the thiol proteins. **(A)** Kyoto Encyclopedia of Genes and Genomes (KEGG) pathway enrichment bubble plot of the identified thiol proteins. **(B)** STRING network analysis of identified thiol proteins.

Biological pathways are functionally united sets of genes that are proven to contribute in specific biological processes, such as energy metabolism. Hence, mapping RMP genes to pathways is an ideal approach to illustrate the roles of RMPs in redox proteome. We mapped RMPs to pathways in KEGG and enriched mapped pathways to explore how RMPs contribute to specific biological processes (Supplementary Table 2). We found that the top 5 significant pathways that RMPs are gathered in are “carbon metabolism” (mgr01200; FDR, 1.17E-6; fold enrichment, 1.74), “biosynthesis of secondary metabolites” (mgr01110; FDR, 1.09E-7; fold enrichment, 1.31), “alanine, aspartate and glutamate metabolism” (mgr00250; FDR, 2.37E-7; fold enrichment, 2.31), “biosynthesis of amino acids” (mgr01230; FDR, 1.92E-5; fold enrichment, 1.50), and “valine, leucine and isoleucine degradation” (mgr00280; FDR, 1.41E-3; fold enrichment, 1.89) (Figure 4A). The top 20 significant pathways included primary metabolisms of carbon metabolism, starch and sucrose metabolism, glycolysis/gluconeogenesis, citrate cycle, galactose metabolism, and pentose phosphate pathway; biosynthesis of secondary metabolites metabolism; purine, pyruvate, and pyrimidine metabolisms; and amino acids metabolism (Figure 4A). These results suggest the wide range functions of redox modifications in primary metabolism, secondary metabolism, as well as nucleotide and amino acid metabolisms of *M. oryzae*.

### Protein Interaction Networks of RMPs

Protein–protein interactions contribute to understanding on cell physiology states, as they play essential roles in almost every process, for instance, signaling transduction. To find interactions among RMPs in *M. oryzae*, a PPI network was built by screening 425 identified RMPs with stringent parameters (Figure 4B). Interestingly, by coloring proteins based on function annotations, we found intricate linkages among RMPs. The important network notes include ribosome, translation initiation, aminoacyl-tRNA biosynthesis, protein processing in ER, and proteasome, indicating that the proteins involved in translation and degradation were enriched. Network notes also include heat shock protein, chaperone, and mitogen-activated protein kinase (MAPK) signaling pathway, suggesting important roles of RMPs in stress and signaling responses. The T-complex protein note was also enriched (Figure 4B). Considering that redox modifications of proteins are important PTMs that affect protein functions, the complicated interactions revealed by the PPI network boost the variety and complexity of biological activities of *M. oryzae*.

### Redox Modified Proteins Involved in Development, Stress Response, and Pathogenicity

**FIGURE 5:**
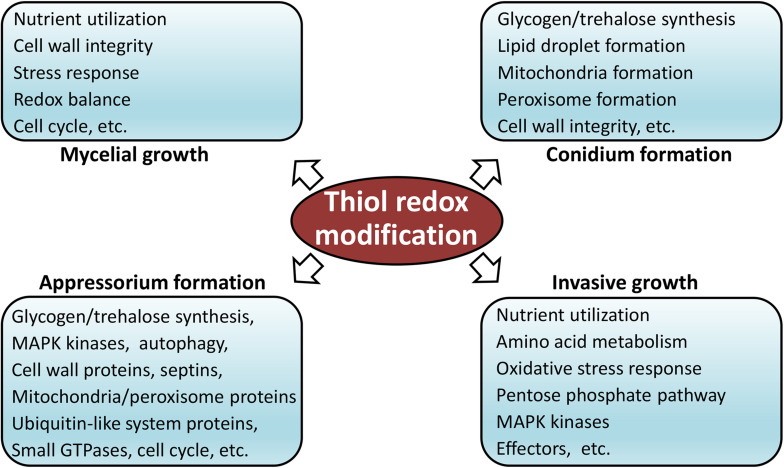
Model of thiol redox-modification-regulated development and infection of *M. oryzae.*

In order to determine functions of redox modification in fungal pathogenesis, we searched the reported *M. oryzae* development, stress response, and pathogenesis-related proteins using all identified RMPs. Notably, as many as 174 proteins that were previously reported as pathogenesis-related proteins were identified to be thiol oxidized in our study. These proteins are associated with a wide range of pathogenesis-related cellular functions, involved in crucial biological process, such as autophagy, ubiquitination, and glycogen metabolism; crucial infection-related pathways, such as MPG1, MagB, GTPases, SLN1, PMK1-MAPK, and MPS1; important subcellular structures, such as septin ring, mitochondria, cytoskeleton, cell wall, and proteasome components; and vital proteins, such as protein kinases and phosphatases. Most of the proteins contained less than three redox sites, while a few also contained more than seven sites, implying a deep involvement of redox modification in pathogenic processes. The complete list of identified redox-modified development or pathogenicity-related proteins is provided in Supplementary Table 3. Using information of these reported thiol redox proteins, we are able to build a model that shows predicted roles of redox modification in the development and infection process of *M. oryzae* (Figure 5). Functions of these reported redox-modified proteins will be discussed in more detail in the following sections.

#### Autophagy

There are eight identified proteins, including cysteine protease ATG4, beclin-1 ATG6, ubiquitin-like protein ATG8, lipase ATG15, sorting nexin-4 ATG24, transmembrane protein ATG27, and DNA binding SaNT domain protein MoSnt2, that were previously demonstrated to be related to autophagy. ATG6, a part of a complex with class III phosphatidylinositol T-kinase (PI3K)/Vps34, has been shown to function in vacuolar protein sorting (VPS) and autophagy in yeast, and the conserved domain also has been identified (Furuya et al., 2005). ATG8 is a member of an evolutionarily conserved ubiquitin-like protein family and can be conjugated to the lipid phosphatidylethanolamine. It is involved in multiple membrane trafficking pathways including autophagy and is required for double membrane-bound structures formation of autophagosomes in *Saccharomyces cerevisiae*. ATG4 is a deconjugation enzyme that can recognize the residues in ATG8 and modulated ATG8 reversibly (Fass et al., 2007; Nakatogawa et al., 2007). ATG15 is required for degradation of autophagic bodies and is essential for the maintenance of lipid droplets amount in the stationary phase in *S. cerevisiae*. The loss of ATG15 leads to enhanced lipolytic activity (Maeda et al., 2015). It has been demonstrated that loss of MoATG24 disrupts mitophagy and consequently results in significantly reduced conidiation. During mitophagy, MoATG24 directly associated with and recruited mitochondria to the autophagic structures (He et al., 2013). ATG27 is required for ATG9 cycling and for specific autophagy in yeast (Yen et al., 2007). MoSnt2 recognizes histone H3 acetylation via its PHD1 domain to recruit the histone deacetylase complex, giving rise to deacetylation of H3 and direct links to MoTor signaling, thus regulating infection-associated autophagy and infection in an epigenetic mechanism. The null mutants of MoSNT2 are impaired in various aspects including infection structure development, conidiation, oxidative stress tolerance, cell wall integrity, autophagy-dependent fungal cell death, and pathogenicity and are compromised in autophagy homeostasis (He et al., 2018).

#### Glycogen Metabolism

Four oxidized proteins, including glycogen debranching enzyme AGL1, glycogen phosphorylase GPH1, glycogen synthase GSN1, alpha, alpha-trehalose-phosphate synthase 1 TPS1, and trehalase TRE1, were reportedly related to glycogen metabolism. AGL1 and GPH1 are required for mobilization of glycogen stores during appressorium development in *M. oryzae*. It has been demonstrated that the deletion mutants of AGL1 and GPH1 showed significant reduction in pathogenicity and in expression of TPS1 and TPS3. TPS1 regulates virulence-associated gene expression in association with Nmr transcriptional inhibitors, by responding to glucose-6-phosphate levels and the balance of NADP/NADPH. GPH1 plays roles in the breakdown of glycogen and is essential in mobilizing glycogen and full virulence. The null mutants of GSN1 significantly reduced the synthesis of intracellular glycogen (Badaruddin et al., 2013). TRE1 locates in the cell wall with characteristics of both neutral and acidic trehalases and is dispensable for pathogenicity, but trehalose degradation is important for the efficient development in plant tissue following initial infection (Foster et al., 2003).

#### Ubiquitin-Like Modification

Seven well-reported ubiquitination-related proteins, including E3 ubiquitin-protein ligase MoBRE1 and MoUBR1, SCF E3 ubiquitin ligase complex F-box protein MoGrr1, E3 ubiquitin ligase complex SCF subunit scon-3 MoSkp1, SUMO protein MoSmt3, SUMO-activating enzyme subunit MoUba2, and ubiquitin carboxyl-terminal hydrolase UBP14, were observed to be redox modified. MoBre1 was shown to be required for growth, conidiation, and pathogenicity in *M. oryzae*, in which its loss resulted in the reduction in di- and tri-methylation level of histone 3 lysine 4 (H3K4). MoUbr1 is essential for conidial adhesion and germination, as it degrades components of cAMP/PKA and MAPK Pmk1 signaling pathways by the N-end rule pathway (Shi et al., 2016). The *Mogrr1* null mutants displayed defects in vegetative growth, melanin pigmentation, conidial production, and resistance to oxidative stress and full virulence and could not generate sufficient turgor pressure in the appressorium for the penetration into plant tissues. The expression of central components of the MAP kinase and cAMP signaling pathways, which are required for appressorium differentiation, was reduced in Mogrr1 mutants (Guo et al., 2015). Skp1 is part of the Skp1-Cullin 1-F-box (SCF) E3 ubiquitin ligase complex, which is necessary for protein degradation, and MoSkp1 is located in spores and germ tubes, with abundant expression in appressoria. The reduction in MoSkp1 results in the decrease in total protein ubiquitination and, thus, defective cell cycle and appressorial development (Prakash et al., 2016). MoSmt3 is a small ubiquitin-related modifier (SUMO) protein present in both the nucleus and cytoplasm, while MoUBA2 is an E1 enzyme present in the nucleus. Nevertheless, both MoSmt3 and MoUba2 are in the nucleus under oxidative stress conditions. Deletion mutants of *MoSMT3* and *MoUBA2* showed significant defects in mycelial growth, conidiation, septum formation, conidial germination, appressorium formation, and pathogenicity, suggesting that they are crucial for infection-related development, stress responses, and pathogenicity in *M. oryzae* (Lim et al., 2018). It has been demonstrated that the ortholog of MoUbp14 plays general roles in ubiquitin-mediated protein degradation in *S. cerevisiae*. Destruction of *MoUBP14* in *M. oryzae* led to loss of pathogenicity and severe reduction in mycelial growth, sporulation, carbon source utilization, and increasement in sensitivity to distinct stresses (Wang et al., 2018).

#### Crucial Infection-Related Pathways

Several well-known proteins in infection-related pathways, such as hydrophobin-like protein MPG1, guanine nucleotide-binding protein subunit alpha MagB, sensor protein SLN1, CMGC/MAPK/ERK protein kinase PMK1, and CMGC/MAPK protein kinase MPS1, were identified as redox-regulated proteins. MPG1 is a small, secreted, cysteine-rich, moderately hydrophobic protein that directs the formation of a rodlet layer on conidia that are interwoven similarly to 5-nm rodlets, thus interacting with hydrophobic surfaces and acting as a developmental sensor for appressorium formation. Deletion mutants of Mpg1 showed reduced efficiency of appressorium formation resulting in impaired pathogenicity (Talbot et al., 1993; Beckerman and Ebbole, 1996; Talbot et al., 1996). MAGB is involved in multiple signal transduction pathways controlling vegetative growth, conidiation, conidium attachment, appressorium formation, mating, and pathogenicity (Liu and Dean, 1997). MoSLN1 acts as a pathogenicity factor that is involved in responses to osmotic stress, the cell wall integrity, and the activity of peroxidases via modulation of intra- and extracellular peroxidase activities. The MoSLN1 mutants displayed hypersensitivity to various stresses, reduced sensitivity to cell wall perturbing agent calcofluor white, and loss of pathogenicity (Zhang et al., 2010). PMK1 is a member of a highly conserved MAP kinase signal transduction pathway, which acts downstream and collaborate with a cAMP signaling pathway for infection structure formation. Disruption of PMK1 leads to loss of the ability to form appressoria invasively in rice plants (Xu and Hamer, 1996). MPS1 is a mitogen-activated protein kinase that is crucial for pathogen penetration, and the mutant of Mps1 showed sensitivity to cell-wall-digesting enzymes, reduced sporulation, and complete loss of pathogenicity resulting from the inability of appressoria to penetrate plant cell surfaces (Xu et al., 1998).

#### GTPases

Redox modification was observed in 11 GTPases, including rho-type GTPase-activating protein LRG1, cell division control protein MgCdc42, rab GDP-dissociation inhibitor MoGdi1, arf GTPase-activating protein MoGlo3, hydrophobin-like protein MPG1, rho-GTPase-activating protein MoRga4 and MoRga5, GTP-binding protein MoRab5B and Rho3, and Ras-like protein Rac1 and MoYpt7. LRG1 was reported as key regulators of infection-associated morphogenesis of *M. oryzae.* Deletion mutants of LRG1 presented significant reduction in hyphal growth, loss of pathogenicity, and decreased sensitivity to cell-wall-perturbing agents (Li et al., 2014). Cdc42 is a member of the Rho-family small GTP-binding proteins and function as a pivotal signaling switch that controls actin cytoskeleton organization and cell polarity, cycling between active GTP-bound and inactive GDP-bound forms. MgCdc42 has been shown to be essential for plant penetration, and disruption of MgCdc42 leads to dramatically reduced pathogenicity resulting from the arrest of penetration and infectious growth due to the defect of turgor and superoxide generation during the appressoria development. Furthermore, the MgCdc42 mutants showed pleotropic defects including gherkin-shaped conidia, delayed germination, and decreased sporulation (Zheng et al., 2009). MoGlo3 is highly expressed during conidiation and early infection stages, which is required for endocytosis, reactive oxygen species scavenging, endoplasmic reticulum (ER) stress response, vegetative growth, conidial production, sexual development, appressorium function, and pathogenicity (Zhang et al., 2017). MoRab5B has been demonstrated to be crucial for vegetative growth and development, conidiogenesis, melanin synthesis, vacuole fusion, endocytosis, sexual reproduction, and plant pathogenesis in *M. oryzae* (Yang et al., 2017). Rac1 takes part in actin cytoskeleton organization and polarized cell growth in many organisms. MgRac1 is required for normal conidial production in amount and morphology, as well as crucial for appressorial formation and pathogenicity (Chen et al., 2008). Rho3 is essential for plant infection and acts as a critical regulator of developmental processes and pathogenicity. Appressoria formed in the absence of Rho3 showed morphological abnormality and defect in plant penetration. Overexpression of MgRho3 contributes to the infectivity of *M. oryzae* (Zheng et al., 2007). Previous study has showed that MoYpt7 is involved in fungal morphogenesis, vacuole fusion, autophagy, stress resistance, and pathogenicity in *M. oryzae*, and the disruption mutants of MoYpt7 exhibited impairment in autophagy, breached cell wall integrity, higher sensitivity to both calcium and heavy metal stress, malformed conidia, defection in appressoria formation, and the inability to cause disease (Liu et al., 2015).

#### Protein Kinases

Ten pathogenesis-related protein kinases were identified as thiol oxidized, including CAMK/CAMK1 protein kinase MoCMK1, CMGC/GSK protein kinase MoGsk1, CMGC/MAPK/P38 protein kinase MoHog1, CMGC/MAPK protein kinase MPS1, CMGC/MAPK/ERK protein kinase PMK1, CAMK/CAMKL/AMPK protein kinase MoSNF1, guanylate kinase MoGuk2, STE/STE7 protein kinase MoMkk1, STE/STE11 protein kinase MST11, and serine/threonine-protein phosphatase MoPpe1. MoCMK1 was shown playing key roles in the pathogenicity of *M. oryzae*, and the MoCMK1 mutants had sparse aerial hyphae, fewer conidia, and a delay in conidial germination and appressorial formation (Liu et al., 2010). The expression of MoGSK1 is regulated by MPS1, another redox-modified MAP kinase, and the deletion of MoGSK1 leads to severe delay in mycelial growth, complete loss of conidiation, and inability to penetrate the host surface by mycelia-formed appressorium-like structures, thus losing the pathogenicity (Zhou et al., 2017). MoSNF1 is critical for sporulation, vegetative growth, and pathogenicity of *M. oryzae*. The MoSNF1 mutant produces less and abnormal conidia and was largely impaired in conidial germination and appressorium formation (Yi et al., 2008). MoGuk2 is involved in the *de novo* GTP biosynthesis pathway and is important for infection-related morphogenesis in *M. oryzae* (Cai et al., 2017). MoMkk1 plays a role in modulating intracellular cAMP levels and response to osmotic stress, and disruption of MoMKK1 resulted in less aerial hypha, defective asexual development, and reduced pathogenicity (Yin et al., 2016). MST11 collaborates with MST7 and PMK1 as a MAP kinase cascade to regulate infection-related morphogenesis (Zhao et al., 2005). MoPpe1 takes part in linking CWI and TOR signaling and is essential for vegetative growth, conidiation, and full virulence (Qian et al., 2018).

#### Subcellular Structures

Proteins located on subcellular structures play various and crucial roles in hyphal growth, conidiogenesis, appressorium development, and pathogenesis of *M. oryzae*, such as cell-wall-related proteins, mitochondrial proteins, and septin proteins. Our data showed that 16 cell-wall-related proteins were redox modified, including chitin deacetylases CDA1, glycosyl hydrolase CDA3, 1,3-beta-glucan synthase component FKS1, alpha-1,3-glucan synthase MoAGS1, tetrahydroxynaphthalene reductase MoBUF1, cell wall biogenesis protein phosphatase SSD1, beta-1,6-galactanase MC63, neutral alpha-glucosidase MoGls2, chitin synthase MgCHS1, MgCHS4, MgCHS5, MgCHS6, and 1,3-beta-glucanosyltransferase Gel1, Gel2, Gel3, and Gel4. Nine mitochondrial proteins were thiol oxidized, including enoyl-CoA hydratase Ech1, peroxisomal hydratase-dehydrogenase-epimerase MFP1, alanine-glyoxylate aminotransferase AGT1, dynamin-A MoDnm1, peroxisome biosynthesis protein MoPEX1, C-8 sterol isomerase ERG2, and D-lactate dehydrogenase MoDLD1, MoDLD2, and MoDLD3. Three septin proteins, eptin-like spn2 MoSep4 and cell division control proteins MoSep5 and MoSep6, were redox regulated. These results revealed the broad roles of redox modification in pathogenicity regulation of *M. oryzae.*

#### Effectors

Effectors play key roles in fungal–plant interactions during fungal invasive growth. Therefore, we also screened the predicted effector proteins according to the previous reports (Ref). In total, 72 thiol-modified sites in 39 putative effectors were identified (Supplementary Table 4). These include several reported effector/elicitor proteins, MoHEG16, MoHrip2, and MoMSP1 (Supplementary Table 3). MoHEG16 is highly expressed in early stage of the invasive hyphae and required full virulence (Mogga et al., 2016). MoHrip2 is an apoplastic effector, which suppresses host immunity and is also required for the full virulence of *M. oryzae* (Nie et al., 2019). The snodprot1 protein MSP1 is another virulence factor in *M. oryzae* (Jeong et al., 2007). These data showed that the effector proteins were also commonly modified by redox modification, suggesting that redox modification could regulate functions of effector proteins for invasion in host cells.

## Discussion

In this study, we systematically explored the landscape of RMPs in *M. oryzae*, which is the first whole redox proteome analysis for rice blast fungus and for fungal pathogen. In total, 1899 proteins with 3713 redox modification sites were identified and studied with different bioinformatic approaches, including motif analysis, domain identification, subcellular localization, and GO and KEGG pathway annotation with corresponding enrichment. An enrichment of lysine residues was found around the cysteine redox modification sites. Regarding localization, the majority (over two-thirds) of redox modifications on proteins occurs in the cytoplasm, mitochondrion, and nucleus. With respect to function annotation and enrichment, RMPs are highly associated with metabolism (e.g., carbon metabolism) and biosynthesis (e.g., secondary metabolites), which is expected. However, the results also show that protein complex (e.g., proteasome and spliceosome, respectively) that evolved in fundamental processes (translation and transcription) can be affected by redox modification.

Redox modification widely exists in both eukaryotes and prokaryotes. The development of high-specificity labeling and enrichment, as well as high-resolution MS techniques, promotes the global thiol redox proteome studies. As early as 2011, redox-sensitive proteome has been investigated, comparing between dormant and non-dormant seeds of wheat (*T. aestivum*). Altogether, 193 reactive Cys were found in 79 unique proteins. The identified proteins are involved in protein synthesis and storage, carbohydrate metabolism, proteases, transport, and signal transduction, which are associated with seed dormancy and protection (Bykova et al., 2011). The redox thiol proteome of *Caenorhabditis elegans* treated with short-term H_2_O_2_ stress was also quantified, and 40 different proteins containing oxidation-sensitive cysteines were identified. These proteins play roles in mobility and feeding protein translation and homeostasis and adenosine triphosphate regeneration (Kumsta et al., 2011). Quantifications of redox thiol proteome were performed on algae, and 80 proteins were identified, which revealed the response to lack of nitrogen by cyanobacterium via post-translational redox changes (McDonagh et al., 2012). In the cyanobacterium *Synechocystis*, approximately 2100 Cys sites from 1060 proteins under light, dark, and photosystem II inhibitor-treated conditions were quantified, implying the broad redox regulation of photosynthetic organisms (Guo et al., 2014). In *P. tricornutum*, the degrees of oxidation of 3845 cysteines were quantified, and 278 redox-sensitive proteins were identified to elucidate the redox-sensitive signaling network (Rosenwasser et al., 2014). McConnell et al. combined the reversibly thiol oxidation and the phosphorylation in proteome quantitative analysis to determine the extent of phosphorylation in the redox thiol proteome in *Chlamydomonas reinhardtii*. A total of 3353 oxidized Cys sites on 1457 enriched proteins were identified, which found the possibility of crosstalk between redox modification and other PTMs (McConnell et al., 2018). Regarding plants, the thiol redox proteome was quantified in *A. thaliana* in 2014 to investigate the oxidative stress after H_2_O_2_ treatment and in 2017 to study the CO_2_ response via bicarbonate treatment. One hundred ninety-five cysteine-containing peptides from 179 proteins and 903 cysteine-containing peptides with 47 significantly changed proteins were identified, respectively. These studies revealed the roles of redox modification in oxidative stress response and CO_2_ response (Liu et al., 2014; Yin et al., 2017). Recently, the thiol redox proteome was quantified in bacteria, *Escherichia coli*, to assess the thiol oxidation status of *E. coli* after being phagocytized by neutrophils. A total of 173 cysteine containing peptides representing 117 proteins were identified, indicating that neutrophil phagocytosis leads to an overall breakdown of the *E. coli* protein thiol homeostasis, which might be a general antimicrobial mechanism for neutrophils to counteract invading bacteria. However, there are no reports on thiol redox proteome of pathogenic fungus, and our knowledge on the global redox modification in pathogenic fungus is still limited.

In order to identify as many redox proteins as possible, acquiring a comprehensive view of the global thiol redox proteome in *M. oryzae*, we employed the samples from all the development stages including mycelia, conidia, appressoria, and infection hyphae. Furthermore, we generated an oxidative stress condition through H_2_O_2_ treatment on mycelia. We identified a large number of redox proteins (1899 proteins) from the mixture described above, contrasted with the number of identified proteins in studies on other species, such as *T. aestivum* (79 proteins), *C. elegans* (40 proteins), cyanobacterium *Prochlorococcus* (80 proteins), cyanobacterium *Synechocystis* (1060 proteins), *P. tricornutum* (278 proteins), *C. reinhardtii* (1457 proteins), *A. thaliana* (179 proteins), and *E. coli* (117 proteins).

Notably, among the redox-regulated proteins identified in the present study, up to approximately 10% (174) proteins are previously reported to be pathogenesis related. These proteins are involved in autophagy, ubiquitination, glycogen metabolism, GTPases, MPS1 pathway, PMK1-MAPK pathway, septin ring, cytoskeleton, cell wall integrity, etc. Autophagy is essential for *M. oryzae* infection, by regulating programmed cell death during appressorium maturation (Veneault-Fourrey et al., 2006) or maintaining lipid body integrity (Liu et al., 2007). Autophagy also regulated carbohydrate catabolism and homeostasis spatially and temporally to ensure successful conidiation in *M. oryzae* (Deng et al., 2009; Deng and Naqvi, 2010). The ubiquitin system modulates protein functions through targeting substrates for ubiquitination and coordinates with cell cycle proteins and controls various cell functions (Prakash et al., 2016; Shi et al., 2016; Lim et al., 2018). The mobilization of glycogen and trehalose is required for appressorium development to fuel biosynthetic processes and turgor generation in the developing appressorium (Thines et al., 2000). Some Rho GTPases interact with each other and regulate signal transduction pathways in eukaryotes. Interestingly, the expression of Rho GTPases MgRac1 was negatively regulated by MgCdc42 and MgRho3, respectively, while all of the three proteins are thiol oxidized, implying a deeply interaction in redox regulation (Zheng et al., 2006). The Pkc1-Mps1 MAP kinase cell wall integrity (CWI) signaling pathway is essential for the production and function of *M. oryzae* appressoria. Disruption of MoMPS1, and terminal MAP kinase of cell wall integrity pathway, leads to appressoria that are unable to penetrate the host plant, thus loss of pathogenicity (Xu et al., 1998; Jeon et al., 2008). The cytoskeleton plays roles in the regulation of multiple cellular processes including but not limited to morphogenesis, cytokinesis, establishment of cell polarity, endocytosis, and exocytosis (Moseley and Goode, 2006; Berepiki et al., 2011). Pore formation at the base of the appressorium is required for the reorganization of the F-actin meditated by a septin ring (Dagdas et al., 2012). In conclusion, these results indicate that redox modification plays an important role in the regulation of pathogenicity in *M. oryzae*.

Interestingly, we found that several pathogenesis-related proteins are able to be regulated by more than a single post-translational modification. It has been reported in *C. reinhardtii* that some of the redox-regulated proteins were found to be additionally phosphorylated (McConnell et al., 2018). In this study, three glycogen metabolism proteins, GPH1, GSN1, and TPS1, and one CMGC/MAPK/ERK protein kinase, PMK1, which are identified as redox modified, can be modified by phosphorylation (Franck et al., 2015). Moreover, GPH1 was reported to be N-glycosylated as well (Chen et al., 2020). Besides GPH1, there are other 14 proteins that can be modified by both redox and N-glycosylation, including the trehalase TRE1, chitin-binding protein CBP1, small GTPases Rac1, translocon channel protein MoSec62 and Sec5, and nine cell-wall-related proteins: MgCHS1, MgCHS4, MgCHS5, MgCHS6, Gel1, Gel2, Gel3, Gel4, and MoGls2 (Chen et al., 2020). Five proteins, including the cell wall biogenesis protein phosphatase SSD1, enoyl-CoA hydratase Ech1, heat shock proteins MoSsb1, isocitrate lyas ICL1, and the key enzymes in the glycolysis pathway, fructose-1,6-bisphosphatase FBP1, can be modified by both redox and succinylation (Wang et al., 2019). One protein, the heat shock proteins MoSsa1, can be modified by both redox and acetylation, with as many as 13 acetylation sites (Liang et al., 2018). The different modifications located on the same pathogenic crucial protein, suggesting a complicated mechanism of post-translational modification in pathogenicity regulation, may be possible by the interaction or crosstalk between different modifications.

The presence of thiol oxidation in the pathogenesis-related proteins indicated a role of redox modification in development, stress response, and pathogenicity of *M. oryzae* (Figure 5). However, further investigation is required for more details on the mechanism of the redox modification on these target proteins. The redox modification during each development stages, especially the infection hypha stages when deep interactions take place between the host and *M. oryzae*.

## Data Availability Statement

The original contributions presented in the study are publicly available. This data can be found here: The mass spectrometry proteomic datasets presented in this study can be found in online repository ProteomeXchange consortium via the PRIDE partner repository with an accession number of px-submission #470462.

## Author Contributions

XZ performed the experiments of sample preparation. X-LC designed the project. All authors performed the data processing and wrote the manuscript.

## Conflict of Interest

The authors declare that the research was conducted in the absence of any commercial or financial relationships that could be construed as a potential conflict of interest.

## Supplementary Material

Supplementary Table 1 List of all thiol redox modified sites and proteins.

Supplementary Table 2 Bioinformatics analyses data of the thiol redox modified proteins.

Supplementary Table 3 Redox-modified pathogenesis-related proteins.

Supplementary Table 4 Thiol redox-modified predicted effector proteins of *M. oryzae*.
